# Antibody-Based Detection and Inhibition of Vaginolysin, the *Gardnerella vaginalis* Cytolysin

**DOI:** 10.1371/journal.pone.0005207

**Published:** 2009-04-16

**Authors:** Tara M. Randis, Ritwij Kulkarni, Jorge L. Aguilar, Adam J. Ratner

**Affiliations:** 1 Department of Pediatrics, Columbia University, New York, New York, United States of America; 2 Department of Microbiology, Columbia University, New York, New York, United States of America; University of Hyderabad, India

## Abstract

Bacterial vaginosis (BV) is the most common vaginal infection worldwide and is associated with significant adverse sequelae. We have recently characterized vaginolysin (VLY), the human-specific cytotoxin produced by *Gardnerella vaginalis* and believed to play a critical role in the pathogenesis of BV and its associated morbidities. We hypothesize that novel antibody-based strategies may be useful for detection of VLY and for inhibition of its toxic effects on human cells. Using purified toxin as an immunogen, we generated polyclonal rabbit immune serum (IS) against VLY. A western blot of *G. vaginalis* lysate was probed with IS and a single band (57 kD) identified. Immunofluorescence techniques using IS detected VLY production by *G. vaginalis*. In addition, we have developed a sandwich ELISA assay capable of VLY quantification at ng/ml concentrations in the supernatant of growing *G. vaginalis.* To investigate the potential inhibitory role of IS on VLY-mediated cell lysis, we exposed human erythrocytes to VLY or VLY pretreated with IS and determined the percent hemolysis. Pretreatment with IS resulted in a significant reduction in VLY-mediated lysis. Similarly, both human cervical carcinoma cells and vaginal epithelial cells exhibited reduced cytolysis following exposure to VLY with IS compared to VLY alone. These results confirm that antibody-based techniques are an effective means of VLY detection. Furthermore, VLY antiserum functions as an inhibitor of VLY–CD59 interaction, mitigating cell lysis. These strategies may have a potential role in the diagnosis and treatment of BV.

## Introduction

Bacterial vaginosis (BV) is the most common vaginal infection worldwide and is associated with significant adverse consequences including and preterm labor and delivery [1], [2], post-partum endometritis [3], and an increased risk of HIV acquisition [4], [5], [6]. Reported prevalence rates range from 10–40% depending upon the population studied [7]. However, suboptimal methods of diagnosis and a high percentage of asymptomatic patients make the true prevalence of BV difficult to ascertain.

The pathogenesis of BV remains poorly understood. It is most commonly defined as a pathological state characterized by the loss of normal vaginal flora, particularly *Lactobacillus* species, and overgrowth of other microbes including *Gardnerella vaginalis*, *Bacteroides* species, *Mobiluncus* species, and *Mycoplasma hominis*. Recent data however, suggest a primary role for *G. vaginalis* as a specific and sexually transmitted etiological agent in BV, as was initially postulated by Gardner and Dukes in 1955 [8], [9], [10].

Our laboratory has recently sequenced and characterized the human-specific, pore-forming toxin produced by *G. vaginalis* known as vaginolysin (VLY) [11]. VLY is a member of the cholesterol-dependent cytolysin (CDC) family of toxins and recognizes the complement regulatory molecule CD59 on the surface of human cells. The VLY-CD59 interaction is believed to play a critical role in the pathogenesis of BV and the development of its associated complications.

We hypothesize that novel antibody-based techniques may be useful for detection and quantification of VLY production. These strategies may represent a substantial improvement in existing methods of BV diagnosis. Furthermore, antibodies generated against VLY may disrupt VLY-CD59 binding, thereby reducing its toxic effects on human cells.

## Materials and Methods

### Ethics statement

The use of human erythrocytes from healthy adult volunteers following verbal informed consent was approved by the Columbia University Institutional Review Board (Protocol IRB-AAAC5641).

### Bacterial strains and cell lines

*G. vaginalis* strains 14018, 14019 and 49145 were purchased from ATCC. ARG3 is a clinical isolate of *G. vaginalis* kindly provided by Susan Whittier. All *G. vaginalis* strains were grown in brain heart infusion supplemented with 10% fetal bovine serum (HyClone), 5% Fildes enrichment (Remel) and 4 ng/ml of amphotericin. Cultures were incubated at 37°C and 5% CO_2_.

Human cell lines were purchased from ATCC. Human cervical endothelial cells (HeLa, ATCC CCL-2) were grown at 37°C and 5% CO_2_ in minimal essential medium (Invitrogen) supplemented with 10% fetal bovine serum and 10 µg/ml ciprofloxacin. Human vaginal endothelial cells (VK2, ATCC CRL-2616) were grown in serum free keratinocyte growth media (Invitrogen) with 0.1 ng/ml EGF, 0.05 mg/ml bovine pituitary extract and 0.4 mM calcium chloride [12].

### Cloning, expression, and purification of VLY

The genomic region encoding VLY was amplified from *G. vaginalis* 14018 as described [11]. Improved purity and greater yield were achieved by generating a truncated construct (excluding the first 50 amino acids from the N-terminal region) using the primer VLY50up (5′- GCCGCC**CATATG**TCGTTGAATAATTATTTGTGG-3′) along with the previously described V6 primer [11]. The PCR product was cloned into the pET28a vector (Novagen), confirmed by sequencing, and transformed into *E. coli* BL21-AI competent cells (Invitrogen) for expression and purification as described [11]. The lytic activity of this truncated recombinant toxoid was unaltered (data not shown).

### Generation of antibodies

Purified VLY toxin was generated and submitted to Cocalico Biologicals (Reamstown, PA). According to their protocol, adult rabbits were injected with a minimum of 100 µg antigen mixed with Complete Freund's Adjuvant subcutaneous and/or intramuscularly at multiple sites. Booster doses containing a minimum of 50 µg antigen mixed with Incomplete Freund's Adjuvant were administered on days 14, 21 and 49. A test bleed was performed on day 56. Prior to the first immunization, serum was collected from each rabbit to serve as negative control.

### Immunofluorescence

*G. vaginalis* 14018 was grown to in culture media and bacterial cells were fixed on a glass chamber slide using 4% paraformaldehyde. Non-specific binding sites were blocked using 5% normal donkey serum and 0.2% triton X-100. Pre-immune or immune serum was added to each slide (1∶500 dilution) for 1 h at room temperature. Following serial washes with PBS and 0.2% triton X-100, donkey anti-rabbit conjugated to Alexa Fluor (AF)-488 (Invitrogen; 1∶1000 dilution) was added for 30 min in the dark with gentle shaking. After washing, chambers were removed from the slide and cover slips were mounted with ProLong Gold antifade with DAPI (Invitrogen). Slides to which no primary antibody was added served as negative controls.

### Western blotting

*G. vaginalis* 14018 was grown on an HBT plate and fresh colonies were resuspended in lysis buffer (BugBuster, EMD Chemicals, Gibbstown, NJ) with benzonase nuclease. The lysate was boiled and separated on a 10% polyacrylamide gel. Proteins were transferred to polyvinylidene difluoride membranes, blocked with 5% milk and probed using rabbit polyclonal anti-VLY antiserum (1∶500,000 dilution). Detection was with HRP-conjugated anti-rabbit IgG (Santa Cruz Biotechnology) and ECL. Membranes probed with pre-immune serum served as a negative control.

### ELISA based assay for VLY production

Four strains of *G. vaginalis* (14018, 14019, 49145, and ARG3) were grown on HBT plates, and colonies were scraped and inoculated into 30 ml of liquid media. A 500 µl aliquot of each culture was obtained every 6 hours for determination of OD_600_. An additional 1 ml sample from each was pelleted by centrifugation and supernatant stored at −20°C prior to ELISA.

Immuno-96 MicroWell plates (Nunc) were coated with anti-pneumolysin antibody (clone 1F11, previously shown to cross-react with VLY) diluted 1∶500 in coating buffer (0.1 M sodium carbonate, pH: 9.5) and incubated at 4°C overnight. Wells were washed with PBS and 0.05% Tween 20. Non-specific binding sites were blocked using PBS with 10% fetal bovine serum for 1 h. Supernatants (100 µl) were added to each well and plates were incubated at room temperature for 2 h. Known concentrations of recombinant VLY toxin diluted in *G. vaginalis* culture media were used as standards. Rabbit polyclonal anti-VLY antiserum (diluted 1∶1000 in blocking solution) was added to each well for 30 min at room temperature. After washing, goat anti-rabbit HRP antibody (Santa Cruz Biotechnology, 1∶1000 dilution) was added for 30 min. Wells were thoroughly washed and 100 µl of TMB substrate (Thermo Scientific) was added to each well and plate was incubated in the dark for 15 min. 50 µl of stop solution (2N sulfuric acid) was added to each well and OD_450_ determined.

### Hemolysis assay

Human blood was obtained by venipuncture, and erythrocytes were immediately isolated by centrifugation and repeated washing in sterile HBSS. A 1% solution of packed erythrocytes in sterile PBS was prepared and added to a 96-well polystyrene V-bottomed plate (100 µl/well). Hemolysis assay was performed as described [11]. Where indicated, toxin was preincubated with pre-immune or immune sera for 30 min at 4°C prior to use in the assay.

### Cytotoxicity assay

24-well plates were seeded with VK2 or HeLa human epithelial cells in appropriate media and grown to >90% confluence. 12 hours prior to use, HeLa cells were weaned from serum. Recombinant VLY toxin diluted in media (10 µg/ml) or vehicle control was added to each well. Where indicated, toxin was preincubated with pre-immune or immune sera for 30 min at 4°C prior to use in the assay. The plates were incubated for 45 min at 37°C and 5% CO_2_. Supernatant was removed and the concentration of lactate dehydrogenase was determined using a commercial kit (Roche) according to the manufacturer's instructions.

### Statistical analysis

Data were expressed as mean±SEM and compared using one-way analysis of variance (ANOVA) with Tukey post-test for comparison of individual groups. (Prism, GraphPad Software)

## Results

### VLY antiserum detects *G. vaginalis* by western blot and immunofluorescence

**Figure 1:**
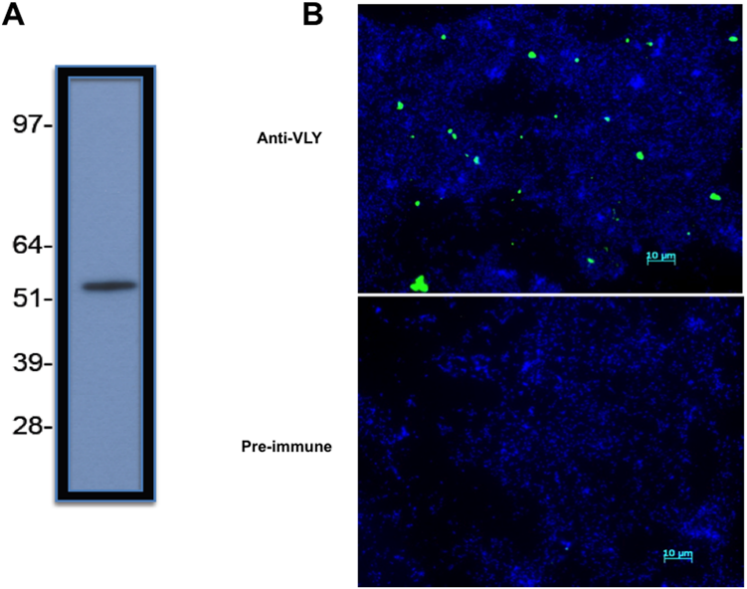
Antibody techniques for the detection of VLY. (A) Western blot of *G.vaginalis* 14018 lysate probed with rabbit polyclonal antiserum (1∶500,000 dilution). Numbers represent approximate MW in kD (B) Immunofluorescent detection of VLY production by *G.vaginalis* using pre-immune rabbit serum (bottom panel) or anti-VLY antiserum (top panel). Anti-rabbit IgG-AF488 was the secondary antibody (green). DNA staining with DAPI demonstrates bacteria in both panels (blue). Scale bar: 10 µm.

Using polyclonal immune serum, we developed novel antibody-based techniques for VLY detection. Western blot analysis of lysed *G. vaginalis* 14018 revealed a single band using polyclonal immune serum as the primary antibody (**Figure 1A**). This corresponds to the predicted 57 kDa molecular mass of VLY and to our prior findings using cross-reacting anti-pneumolysin antibody [11]. There were no visible bands detected on membranes probed with pre-immune serum and processed identically (data not shown). Immunofluorescent detection of VLY associated with whole *G. vaginalis* was detected microscopically using immune serum and fluorescently labeled anti-rabbit secondary antibodies (**Figure 1B**). Preimmune serum did not lead to detectable fluorescence of *G. vaginalis* (**Figure 1B**).

### ELISA detects VLY production by *G. vaginalis*

**Figure 2:**
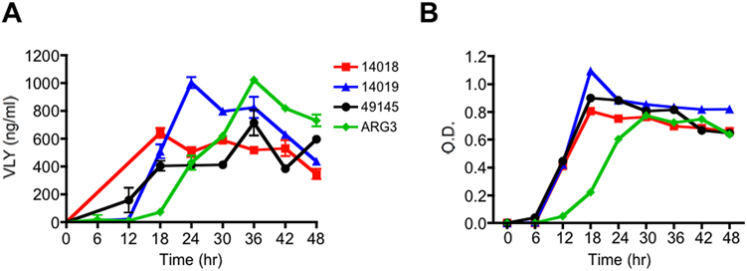
Quantification of VLY production in vivo. (A) Detection of VLY in supernatants from four different strains of *G. vaginalis* supernatants by ELISA at various time points following inoculation of broth culture. (B) Bacterial growth (OD_600_) over the course of the experiment.

Utilizing polyclonal immune serum, we developed a sandwich ELISA capable of quantifying VLY production in vivo. We used a cross-reacting monoclonal anti-pneumolysin antibody as for capture and polyclonal anti-VLY for detection. Four strains of *G. vaginalis* (including three well characterized laboratory strains and one clinical isolate) were inoculated into liquid media and bacterial growth curves were generated as determined by optical density (600 nm). All strains of *G. vaginalis* grew at similar rates in liquid media (**Figure 2B**). We assessed in vivo toxin production at regular intervals (every 6 h) during growth. The sensitivity of the assay was at the level of ng/ml concentrations in bacterial supernatants. VLY production peaked at 24–36 hr in all four strains and correlated with bacterial concentration (**Figure 2A**).

### Antiserum against VLY inhibits toxin-mediated cytolysis

**Figure 3:**
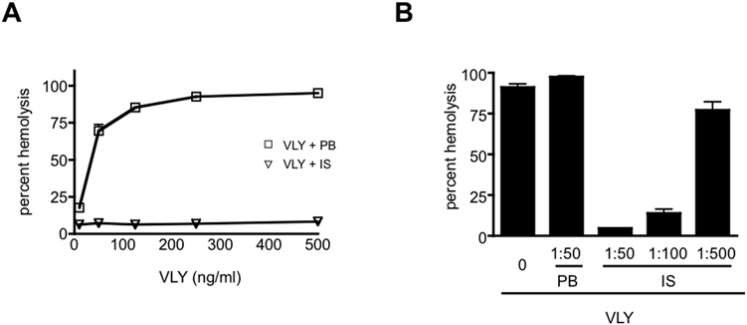
Polyclonal immune serum inhibits VLY-mediated hemolysis. (A) Human erythrocytes were exposed to varying concentrations of purified recombinant VLY for 30 min. Cells were pelleted, and hemoglobin release was determined by OD_415_ of the supernatant. Values were normalized to 100% lysis. When indicated, VLY was preincubated with pre-bleed (VLY+PB) or immune serum (VLY+IS) diluted 1∶50 for 30 min prior to use in the assay. (B) Erythrocytes were exposed to VLY (500 ng/ml), VLY+PB, or serial dilutions of VLY+IS (P<0.01 for VLY+PB versus all VLY+IS dilutions).

Human erythrocytes are susceptible to VLY-mediated hemolysis at low ng/ml concentrations (**Figure 3A**). We hypothesized that antiserum would neutralize VLY, mitigating its cytolytic activity. In order to test this hypothesis, VLY was pre-incubated with polyclonal immune serum (1∶50 dilution) for 30 minutes prior to exposure to human erythrocytes. Pre-incubation with immune serum resulted in significantly less hemolysis compared to cells exposed to untreated or pre-immune serum-treated VLY (**Figure 3A**). Inhibition of VLY-mediated lysis by immune serum was dose-dependent (**Figure 3B**) and was significant even at the highest (1∶500) dilution.

**Figure 4:**
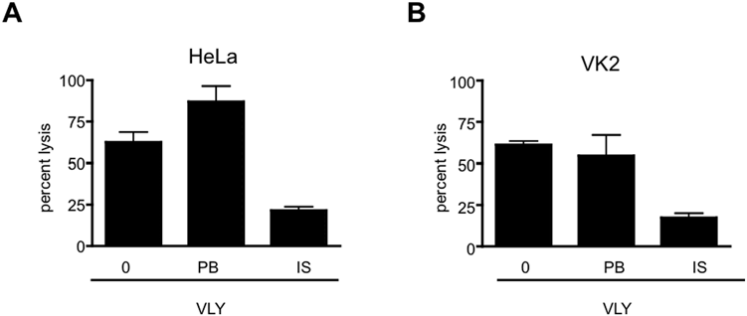
Immune serum inhibits VLY-mediated lysis of human cervical and vaginal cells. Human cervical (A, HeLa) or vaginal (B, VK2) epithelial cells were exposed to VLY (10 µg/ml), VLY+PB, or VLY+IS. Lysis was measured by LDH release assay following 30 min of incubation with toxin. Values were normalized to 100% lysis for each cell line (P<0.05 for VLY+IS versus VLY+PB for HeLa and VK2 cell lines).

The cytolytic activity of VLY in the lower genital tract is believed to be critical for the development of bacterial vaginosis and its associated morbidities. Inhibition of VLY-mediated cell lysis in these tissues may therefore, have important physiologic relevance. Using an LDH assay, we assessed the cytolytic activity of VLY in human cervical epithelial cell (HeLa) and vaginal epithelial cells (VK2). VLY-mediated lysis of both HeLa cells (**Figure 4A**) and VK2 cells (**Figure 4B**) was markedly reduced in the setting of immune serum.

## Discussion

Existing methods of diagnosis for BV are suboptimal and frequently underutilized by practitioners. A recent study by Hogan et al. reports that the prevalence of BV among pregnant women varies greatly depending on the diagnostic criteria used [13]. Furthermore, the authors conclude that the methodology employed by most physicians would understate the true prevalence of BV.

Established in 1983, Amsel's criteria are widely accepted as the best available means for diagnosing BV in the clinical setting and require at least three of the following conditions be present: vaginal discharge, amine odor, pH>4.5 and the presence of clue cells [14]. These criteria are complex, somewhat subjective, and necessitate that microscopy equipment be present on site. Moreover, because the vast majority of women with BV are asymptomatic, application of these criteria may be impractical. A study by Keane et al. noted that the Amsel criteria were used by only 65% of clinics in the UK and only 31% of the practitioners utilized all four criteria in their assessment [15].

The Nugent scoring system for interpretation of Gram-stained vaginal smears was put forth in an attempt to standardize diagnosis of BV and increase inter-rater reliability [16]. Scores are assigned to Gram-stained vaginal smears according to the number of specific bacterial morphotypes seen per microscopic 1000× visual field. While the Nugent scoring system exhibits superior sensitivity and specificity compared to the Amsel criteria [17], its use remains largely restricted to research protocols. Furthermore, questions regarding the risk of potential morbidities and the need for antimicrobial therapy in those women found to have “intermediate flora” remain unanswered [18], [19].

The sheer prevalence of BV and its associated morbidities justify the exploration and development of improved diagnostic strategies easily incorporated into diverse clinical settings. Several alternative diagnostic methodologies focusing upon the detection of microbial virulence factors produced by the various BV-associated organisms have been proposed in recent years. These include detection of bacterial sialidases, determination of amine levels, and measurement of proline aminopeptidase activity [20], [21], [22]. While these techniques are relatively simple, rapid and inexpensive, they fail to identify the specific microbial pathogens present.

A potential role for novel, molecular based techniques for the diagnosis of BV has recently emerged. Importantly, preliminary studies evaluating these PCR-based strategies have provided additional evidence for *G. vaginalis* as the primary etiologic agent of BV [23], [24], [25]. Menard et al. analyzed 213 vaginal samples from pregnant women using a molecular probes targeting 8 BV–related organisms [24]. These authors report that an increased load of *G. vaginalis* (>10^9^ copies of *G. vaginalis* DNA per ml) had both high negative and positive predictive values for the diagnosis of BV. While these molecular based diagnostic strategies are promising, the required expertise, laboratory resources and expense limits their use in the primary care setting.

We demonstrate here that antibody-based techniques are an effective means of identifying *G. vaginalis* through the detection of its pore-forming toxin VLY. The ELISA based assay in particular, is sensitive, robust and directly correlates with the concentration of *G. vaginalis*, reported to be an independent predictor of BV and subsequent preterm delivery [23], [24], [25], [26]. In addition to the diagnostic utility of these antibody-based strategies, they may have an additional role in the treatment of BV. We demonstrated antibody-mediated inhibition of lysis of erythrocytes as well as the likely target cell of VLY in vivo, female genital tract epithelial cells. While successful eradication of BV in pregnant women is possible utilizing appropriate antimicrobial therapy, many women exhibit persistent symptoms, recurrent disease, and persistence of abnormal vaginal flora. Furthermore, several large clinical trials have demonstrated that the use of antibiotics in these women has not been associated with a reduction in preterm birth [27], [28]. The failure of antimicrobial therapy to reduce BV-associated preterm labor may be attributable its inability to mitigate the resultant inflammatory cascade already underway.

The human-restricted activity of VLY represents a barrier to the study of pathogenesis and candidate therapeutic strategies. Disruption of the interaction of VLY with its host cell receptor, human CD59, may represent a novel approach to the treatment of BV. We demonstrated that polyclonal immune serum functions to inhibit the VLY-CD59 interaction, thereby reducing its toxic effects on a variety of human cell lines. These finding may serve as a preliminary basis for in vivo studies investigating a potential role for immunotherapy in the management of women with BV and the development of vaccine based strategies for disease prevention.
